# Modeling and Performance Analysis of Large-Scale Backscatter Communication Networks with Directional Antennas

**DOI:** 10.3390/s22197260

**Published:** 2022-09-25

**Authors:** Qiu Wang, Yong Zhou

**Affiliations:** School of Computer Science and Technology, China University of Mining and Technology, Xuzhou 221000, China

**Keywords:** backscatter communications, large-scale network, stochastic geometry, directional antennas, connectivity, spatial throughput

## Abstract

Backscatter communication (BackCom) constitutes intriguing technology that enables low-power devices in transmitting signals by reflecting ambient radio frequency (RF) signals that consume ultra-low energy. Applying the BackCom technique in large-scale networks with massive low-power devices can effectively address the energy issue observed in low-power devices. Prior studies only consider large-scale BackCom networks equipped with omni-directional antennas, called Omn-BackCom Net. To improve the network’s performance, we employ directional antennas in large-scale BackCom networks, called Dir-BackCom Nets. This article establishes a theoretical model for analyzing the performance of Dir-BackCom Nets. The performance metrics include both connectivity and spatial throughput. Our model is genaralized for both Dir-BackCom Nets and Omn-BackCom Net. The accuracy of our theoretical model is verified by extensive simulations. Results indicate that Dir-BackCom Nets can improve connectivity and spatial throughput. Moreover, results show that the throughput can be maximized by choosing an optimal density of BTs. In addition, both the connectivity and spatial throughput of BackCom Nets can be improved by choosing a directional antenna with a proper beamwidth and gain of the main lobe. Our theoretical model and results can offer beneficial implications for constructing Dir-BackCom Nets.

## 1. Introduction

In the era of the Internet of things (IoT), there is an expectation that massive low-power devices will be used in large-scale IoT networks to monitor, sense, and generate information to support various IoT applications such as smart city/factory/agriculture [1,2,3,4,5]. Powering such massive devices is challenging. As a promising technique, the backscatter communication (BackCom) technique enables devices to transmit data by reflecting environmental radio frequency (RF) signals with consuming ultra-low energy. Therefore, deploying the BackCom technique into large-scale IoT networks can effectively overcome the battery issue of low-power devices [6].

In large-scale BackCom networks (BackCom Nets), backscatter transmitters (BTs) need to transmit data by reflecting RF signals from ambient energy/signal providers: carrier emitters (CEs) [7]. Consequently, the transmission of BTs is dependent on the location relationship between BTs and CEs. As a result, the communication performance of BackCom is heavily dependent on the location distribution of both CEs and BTs [6,7,8]. Therefore, it is necessary to investigate the performance of large-scale BackCom Net and analyze the relationship between the distribution of both CEs and BTs and the performance of BackCom Nets.

In the performance analysis of BackCom networks, because CEs and BTs are usually distributed with randomness in large-scale networks [6,7,8,9,10], both the energy received at BTs and interference received at a backscatter receiver (BR) are usually variables. A popular approach for analyzing the energy/interference is to utilize a mathematical tool known as stochastic geometry [11,12]. Assisted with stochastic geometry, the energy and interference can be analyzed according to the spatial distribution of CEs and BTs, respectively [6,8]. Based on stochastic geometry, ref. [9] first proposed a theoretical framework to analyze the performance of large-scale BackCom Nets. Then, many researchers analyzed large-scale BackCom Nets in various network environments by combining different techniques [6,7,8,13,14,15]. Specifically, ref. [8] analyzed BackCom Nets by combining wireless power transfer (WPT). Ref. [13] analyzed the performance of large-scale BackCom Nets based on radio frequency identification (RFID) communications. Then, studies [14,15] analyze the performance of cognitive BackCom Nets. More recently, ref. [6] analyzes the performance of cellular BackCom Nets. However, in the above-mentioned studies, the performance analysis only focuses on the network where BTs and BRs are mounted with omni-directional antennas, where omni-directional antennas transmit/receive signals equally in a plane [16]. The full-direction coverage in a plane of omni-directional antennas can play a great role in applications in which devices need to sense/transmit data in all surrounding directions. However, in applications where devices sense/transmit data by peer-to-peer communications, omni-directional antennas will suffer from poor communication performance because of the interference in some undesired directions and short transmission ranges [17].

Different from omni-directional antennas, directional antennas, assisted by beamforming technique, can enhance signals transmission/reception in intended directions and can simultaneously decrease signal transmission/reception in other directions. In peer-to-peer communication applications, due to the beamforming transmission, directional antennas can decrease interferences in undesired directions, thereby potentially improving the connectivity and throughput [18]. Moreover, the high gain of directional antennas can compensate the heavy path loss of double-path of backscatter communications (i.e., BT receive RF signals first and then reflect signals). Therefore, applying directional antennas to a large-scale BackCom Net can potentially improve network performance. In addition, considering that directional antennas have been widely deployed in 5G communication devices and WiFi devices with 802.11ad to compensate for the high attenuation of the path loss of MMwave [19,20,21], it is worth developing a theoretical model to analyze the performance of large-scale BackCom Nets in which BTs and BRs are equipped with directional antennas, which are called Dir-BackCom Nets.

**Figure 1:**
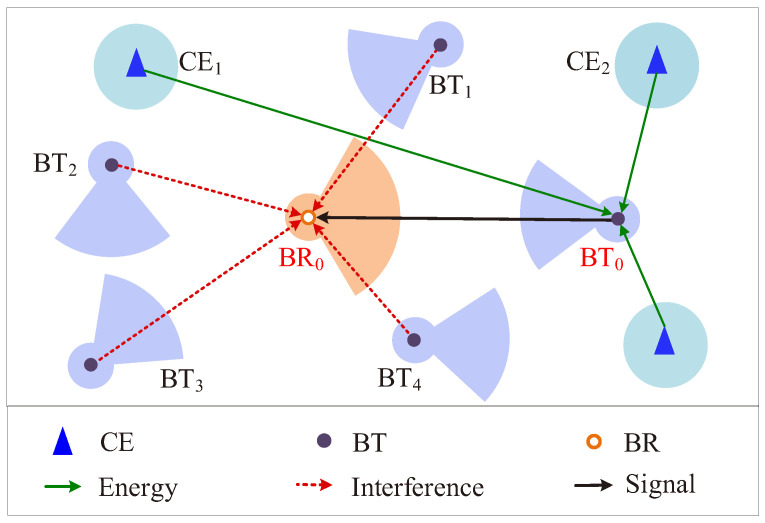
Example of a part of a Dir-BackCom Net.

To the best of our knowledge, there is no study that analyzes the performance of large-scale Dir-BackCom Nets. The performance analysis of large-scale Dir-BackCom Nets is far more complex than large-scale BackCom Nets mounted with omni-directional antennas, called Omn-BackCom Nets. This is because the transmission/reception strength of a directional antenna varies in different directions (i.e., it is strong in some directions and weak in some directions), leading to the fact that the received energy/interference at a directional antenna in a large-scale network is spatially inhomogeneous. For example, Figure 1 illustrates a part of a Dir-BackCom Net around a transmission pair BT_0_-BR_0_, where BT_0_ receives the energy (i.e., RF signals) provided from CEs and then reflects signals to BR_0_. We can observe that BT_0_ receives energy from CE_1_ and CE_2_ by different antenna gains, thereby leading to inhomogeneous energy reception in the space around BT_0_. Then, BR_0_ receives interference by different antenna gains (i.e., it receives interference from BT_1_ and BT_4_ by a high gain and receives interference from BT_2_ and BT_3_ by a low gain). In addition, the interference from BT_1_ and BT_3_ is transmitted by a high gain, while the interference from BT_2_ and BT_4_ is transmitted by a low gain. As a result, the received interference at BR_0_ from all BT_1_ to BT_4_ is different from each other, resulting in the fact that the interference received at BR_0_ is more spatially inhomogeneous. Therefore, compared with Omn-BackCom Nets where the received energy/interference is spatially homogeneous, the complexity of the performance analysis in Dir-BackCom Nets significantly increased.

Therefore, the main goal of this paper is to develop a theoretical model to analyze the performance of Dir-BackCom Nets. The main contributions are summarised as follows.

- In contrast to prior related studies focusing on the performance of Omn-BackCom Nets, we first identify Dir-BackCom Nets. Then, we establish a theoretical model to analyze the performance of Dir-BackCom Nets. This model is general for BackCom Nets equipped with omn-directional antennas or directional antennas.
- We derive both the connectivity and the spatial throughput of Dir-BackCom Nets. Results indicate that the spatial throughput can be maximized by selecting an optimal density of BTs.
- We make a comparison of the performance among Dir-BackCom Nets, Omn-BackCom Nets, and networks in which BTs and BRs are equipped with directional antennas and omni-directional antennas, respectively, called Dir-Omn-BackCom Nets. Our results indicate that equipping directional antennas at BTs or BR can improve network performance. Moreover, we perform a comparison of performance among Dir-BackCom Nets equipped with different directional antennas with varied antenna beamwidths and antenna gains. Results show that the performance of Dir-BackCom Nets can be improved by choosing suitable directional antennas with proper antenna beamwidths and gains of the main lobe.

The rest of the article is organized as follows: Section 2 presents system models. Then, we analyze the connectivity of Dir-BackCom Nets in Section 3. Next, Section 4 derives the network throughput of Dir-BackCom Nets. The simulation and numerical results are presented in Section 5. Finally, the article is concluded in Section 6.

## 2. System Models

### 2.1. Network Model

**Figure 2:**
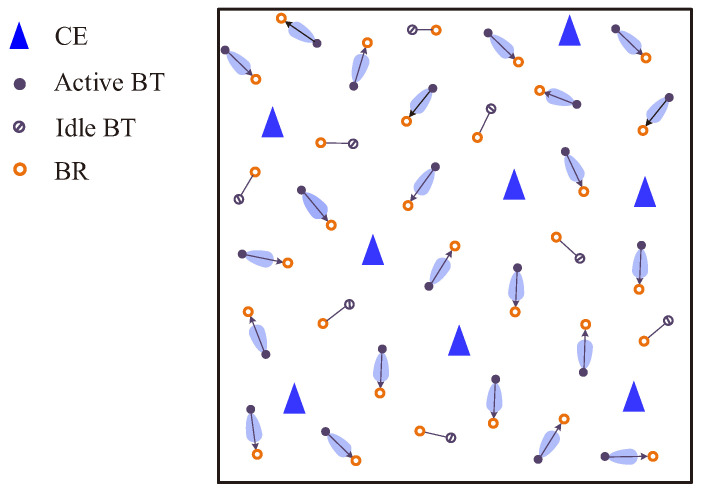
Large-scale Dir-BackCom Net.

We consider a large-scale Dir-BackCom network as shown in Figure 2. In the network, CEs follow a homogeneous Poisson point process (HPPP) denoted by $\Theta_{\mathrm{CE}}=\left\{{\mathrm{CE}}_{i},i=1,2,\ldots \right\}$ with density $\lambda_{\mathrm{CE}}$; BTs follow another independent HPPP denoted by $\Theta_{\mathrm{BT}}=\left\{{\mathrm{BT}}_{j},j=1,2,\ldots \right\}$ with density $\lambda_{\mathrm{BT}}$, where $\lambda_{\mathrm{BT}}\gg \lambda_{\mathrm{CE}}$ [6]. Then, we consider that each BT is associated with a BR, where the location orientation of the BR is uniformly distributed in [0, 2π] around its paired BT [18].

We assume that each BT/BR is mounted with a directional antenna, where the orientation of the directional antenna of a BT/BR is directed towards its paired BR/BT [18]. As a result, both orientations of the directional antennas of BTs and BRs are uniformly distributed in [0,2π]. In addition, we consider that each CE is mounted with an omni-directional antenna to provide RF signals for all BTs [6]. We assume that a BT can receive RF signals from all CEs as energy [6]. When the received energy at a BT is higher than a threshold, BT can be activated from an idle state and conduct backscatter communications.

It is worth noting that our performance analyses do not consider the devices near network border, because their performance analysis is quite complex due to the fact that their performance is related to the specific shape of the network’s edge [22]. Therefore, similarly to [6,7,8,9,10,15,18], this article only considers the communication performance of devices that are not affected by border effects.

### 2.2. Channel Model

In the large-scale Dir-BackCom Net, multiple orthogonal frequency channels can be allocated to CEs. Because available frequency channels are limited while there are massive CEs in the large-scale network, multiple CEs reuse an identical frequency channel. Assume that the number of available frequency channels is *N*. Then, the density of co-channel CEs is $\lambda_{\mathrm{CE}}/N$. In addition, we assume that BTs can randomly choose one frequency channel to receive RF signals from CEs. Consequently, the density of co-channel BTs is $\lambda_{\mathrm{BT}}/N$.

During propagation, we consider a typical propagation model $r^{-\alpha }hG_{t}G_{r}$, where *r* is the propagation distance, α is the path loss exponent with 2<α<6 [18], *h* is the small-scale fading factor following an exponential distribution with mean 1 [6,18], and $G_{t}$ and $G_{r}$ are the antenna gains of the transmitters and receivers, respectively.

### 2.3. Antenna Model

**Figure 3:**
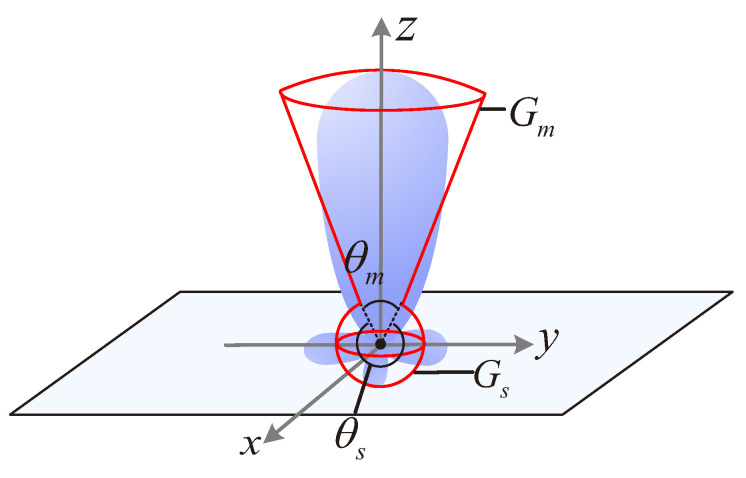
Realistic directional antenna and keyhole antenna model.

The antenna gain of an omni-directional antenna, denoted by $G_{o}$, is $G_{o}=1$. The radiation pattern of a realistic directional antenna is shown in Figure 3 [16,23,24,25], which comprises one main lobe and several side/back lobes. We can observe that each lobe of the radiation pattern varies in directions. This leads to the intractability of the performance analysis in *large-scale* BackCom Nets. For analytical tractability, similarly to [17,26,27,28], we simplify the realistic directional antenna to a keyhole antenna model, as shown in Figure 3. The radiation pattern of the keyhole antenna model consists of a main lobe with gain $G_{m}$ in beamwidth $\theta_{m}$ and a constant side lobe with gain $G_{s}$ in beamwidth $\theta_{s}$. The antenna gain of the keyhole antenna model, denoted by $G_{d}$, can be described by the following equation:

(1) $$G_{d}\left(\theta \right)=\left\{\begin{array}{cc} G_{m} & \theta \in \left(0,\;\theta_{m}\right)\left(\theta \mathrm{within}\theta_{m}\right)\\ G_{s}=\frac{2-G_{m}\left(1-\cos\left(\frac{\theta_{m}}{2}\right)\right)}{\cos\frac{\theta_{m}}{2}-1} & \theta \in \left(\theta_{m},2\pi \right)\left(\theta \mathrm{within}\theta_{s}\right), \end{array}\right.$$

where the detailed derivation process of the antenna gain is provided in Appendix A.

To assure the generality of our model, we consider that directional antennas equipped by BTs and BRs can have different values of both $G_{m}$ and $\theta_{m}$. Therefore, we let $\theta_{\mathrm{tm}}$ and $\theta_{\mathrm{ts}}$ denote the beamwidth of the main lobe and the side lobe of the antenna equipped by BTs, respectively. Then, let $G_{\mathrm{tm}}$ and $G_{\mathrm{ts}}$ denote the corresponding gains within $\theta_{\mathrm{tm}}$ and $\theta_{\mathrm{ts}}$, respectively. Similarly, we let $\theta_{\mathrm{rm}}$ and $\theta_{\mathrm{rs}}$ denote the beamwidth of the main lobe and the side lobe of the antenna equipped by a BR, respectively. Then, let $G_{\mathrm{rm}}$ and $G_{\mathrm{rs}}$ denote the corresponding gains within $\theta_{\mathrm{rm}}$ and $\theta_{\mathrm{rs}}$, respectively.

### 2.4. Backscatter Communication Model

**Figure 4:**
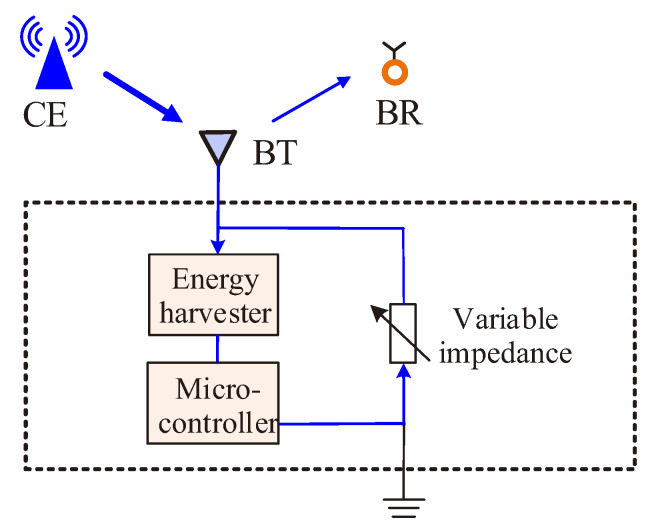
Backscatter communication scheme.

Figure 4 shows the adopted backscatter communications model [6,8,9]. RF signals generated from CEs are used for both energy harvesting and signal transmission at BTs. Specifically, on the one hand, RF signals are harvested as energy at BTs to run the circuits of backscatter communications; on the other hand, RF signals are reflected to BRs for information transmission. The reflected signals are modulated by a micro-controller: RF signals are reflected in different power levels to stand for various bits when the micro-controller switches to various impedances [29]. The ratio of the reflected power to the total received power at BTs is named as *reflection coefficient* and is denoted by η. Assume that the received power at a BT is denoted by $P_{\mathrm{BT}}^{r}$, the reflected power at BTs, denoted by $P_{\mathrm{BT}}^{t}$, and the power for energy harvesting at BTs, denoted by $P_{\mathrm{BT}}^{h}$, can be expressed as the following two equations.

(2) $$P_{\mathrm{BT}}^{t}=\eta P_{\mathrm{BT}}^{r}.$$

(3) $$P_{\mathrm{BT}}^{h}=\left(1-\eta \right)P_{\mathrm{BT}}^{r}.$$

RF signals used for energy harvesting can be stored at BTs by a energy harvester. Following [30,31,32], we adopt a non-linear energy harvesting model proposed in [33]. The harvested energy, denoted by $P_{e}\left(P_{\mathrm{BT}}^{h}\right)$, can be expressed as follows:

(4) $$\begin{array}{c} P_{e}\left(P_{\mathrm{BT}}^{h}\right)=\frac{\Omega \left(P_{\mathrm{BT}}^{h}\right)-E_{\max}C}{1-C},\;\;C=\frac{1}{1+\exp(c_{1}c_{2})}, \end{array}$$

where $E_{\max}$ is the saturated energy power that can be stored at the energy harvester maximally; *C* is a constant to ensure a zero-input or zero-output response at the energy harvester, $c_{1}$ and $c_{2}$ are two constants related to the specific capacitance, resistance, and diode turn-on voltage in energy harvesting circuits. The term $\Omega (P_{\mathrm{BT}}^{h})$ is a function of $P_{\mathrm{BT}}^{h}$, which is given by the following.

(5) $$\begin{array}{c} \Omega \left(P_{\mathrm{BT}}^{h}\right)=\frac{E_{\max}}{1+\exp(-c_{1}\left(P_{\mathrm{BT}}^{h}-c_{2}\right))}. \end{array}$$

The harvested energy of BTs ($P_{e}\left(P_{\mathrm{BT}}^{h}\right)$) can be used to activate backscatter communications. Let $P_{c}$ denote the power threshold for activating the circuits. Then, the trigger condition for backscatter communications is provided by the following.

(6) $$\begin{array}{c} P_{e}\left(P_{\mathrm{BT}}^{h}\right)\geq P_{c}. \end{array}$$

## 3. Connectivity

The connectivity of BackCom networks is defined as follows:

**Definition** **1.** *The connectivity is the probability that a BT-BR pair can establish a link successfully.*

The condition of a successful link between a BT-BR pair is as follows:

1. The BT can receive sufficient power to activate backscatter communications.
2. The signal reflected from the BT can be successfully received by the BR.

We call the probability that a BT can be activated as the active probability. We first analyze the active probability in Section 3.1. Then, we analyze the connectivity in Section 3.2.

### 3.1. Active Probability

The backscatter communications at a BT can be activated when the harvested energy of the BT is higher than a threshold $P_{c}$ (i.e., $P_{e}\geq P_{c}$). Since the harvested energy at a BT is dependent on the received power at a BT, we first analyze the received power at a BT (i.e., $P_{\mathrm{BT}}^{r}$).

**Figure 5:**
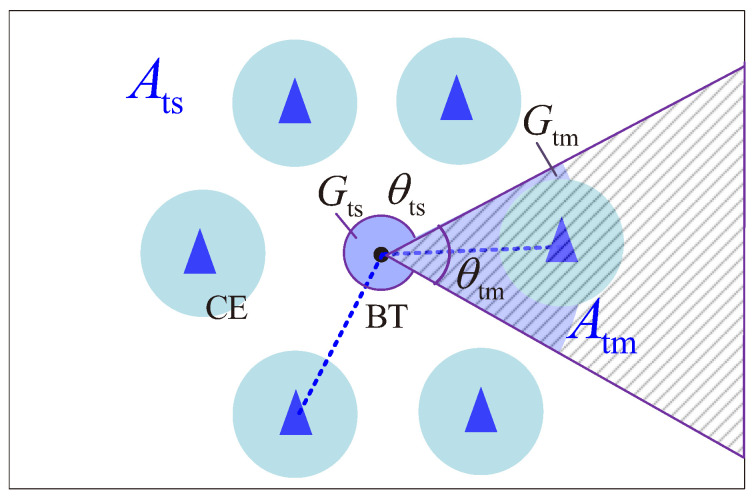
Example to illustrate the energy reception at a BT.

Figure 5 provides an example for illustrating the energy reception at a BT. We can observe that a BT receives energy by both the main lobe within $\theta_{\mathrm{tm}}$ and the side lobe within $\theta_{\mathrm{tm}}$. Therefore, we divide the surrounding space of a BT into two regions:

- The region in which a BT receives signals by the main lobe (the shadowed region in Figure 5), named region $A_{\mathrm{tm}}$.
- The region in which a BT receives signals by the side lobe, named region $A_{\mathrm{ts}}$.

Let $P_{\mathrm{BT}}^{A_{\mathrm{tm}}}$ and $P_{\mathrm{BT}}^{A_{\mathrm{ts}}}$ denote the received power at a BT in regions $A_{\mathrm{tm}}$ and $A_{\mathrm{ts}}$, respectively. The total received power at a BT can be given by $P_{\mathrm{BT}}^{r}=P_{\mathrm{BT}}^{A_{\mathrm{tm}}}+P_{\mathrm{BT}}^{A_{\mathrm{ts}}}$. We let $\Theta_{\mathrm{CE}}\left(A_{\mathrm{tm}}\right)$ and $\Theta_{\mathrm{CE}}\left(A_{\mathrm{ts}}\right)$ denote the HPPPs of CEs providing energy for a BT in regions $A_{\mathrm{tm}}$ and $A_{\mathrm{ts}}$, respectively, where $\Theta_{\mathrm{CE}}\left(A_{\mathrm{tm}}\right)$ and $\Theta_{\mathrm{CE}}\left(A_{\mathrm{ts}}\right)$ are mutually independently. If a PPP is distributed in a region, the PPPs distributed in non-lapping sub-regions are mutually independent [34]; let $P_{\mathrm{CE}}$ denote the transmitted power of CEs. Then, the power of received energy at a BT can be provided by the following:

(7) $$\begin{array}{c} P_{\mathrm{BT}}^{r}=P_{\mathrm{BT}}^{A_{\mathrm{tm}}}+P_{\mathrm{BT}}^{A_{\mathrm{ts}}}=\sum_{{\mathrm{CE}}_{i}\in \Theta_{\mathrm{CE}}\left(A_{\mathrm{tm}}\right)}P_{\mathrm{CE}}r_{i}^{-\alpha }h_{i}G_{\mathrm{tm}}+\sum_{{\mathrm{CE}}_{i}\in \Theta_{\mathrm{CE}}\left(A_{\mathrm{ts}}\right)}P_{\mathrm{CE}}r_{i}^{-\alpha }h_{i}G_{\mathrm{ts}}, \end{array}$$

where $r_{i}$ is the distance between CE_*i*_ and the studied BT, $h_{i}$ is the small-scale fading factor for the path between CE_*i*_ and the studied BT, and $G_{\mathrm{ts}}=\frac{2-G_{\mathrm{tm}}\left(1-\cos\left(\frac{\theta_{\mathrm{tm}}}{2}\right)\right)}{\cos\frac{\theta_{\mathrm{tm}}}{2}-1}$.

Then, we provide both the cumulative distribution function (CDF) and the probability density function (PDF) of the received power at a BT ($P_{\mathrm{BT}}^{r}$) as the following proposition.

**Proposition** **1.** *The CDF and the PDF of the received power at a BT ($P_{BT}^{r}$), denoted by $F_{P_{BT}^{r}}\left(x\right)$ and $f_{P_{BT}^{r}}\left(x\right)$, respectively, are given by the following:*

(8) $$\begin{array}{c} F_{P_{BT}^{r}}\left(x\right)=1-\frac{\alpha }{2\pi }\int_{0}^{\infty }\frac{\sin(\gamma \sin\frac{2\pi }{\alpha })}{\gamma \exp\left({\left(\frac{\gamma }{W}\right)}^{\frac{\alpha }{2}}x+\gamma \cos\frac{2\pi }{\alpha }\right)}d\gamma , \end{array}$$

(9) $$\begin{array}{c} f_{P_{BT}^{r}}\left(x\right)=\frac{\alpha }{2\pi }\int_{0}^{\infty }\frac{\sin\left(\gamma \sin\frac{2\pi }{\alpha }\right){\left(\frac{\gamma }{W}\right)}^{\frac{\alpha }{2}}}{\gamma \exp\left({\left(\frac{\gamma }{W}\right)}^{\frac{\alpha }{2}}x+\gamma \cos\frac{2\pi }{\alpha }\right)}d\gamma , \end{array}$$

*where the following is the case. $W=\frac{\pi \lambda_{CE}P_{CE}^{\frac{2}{\alpha }}\left(\theta_{tm}G_{tm}^{\frac{2}{\alpha }}+\theta_{ts}G_{ts}^{\frac{2}{\alpha }}\right)}{N\alpha \sin\left(\frac{2\pi }{\alpha }\right)}$.*

**Proof.**  The proof is given in Appendix B. □

Then, we provide the active probability, denoted by $P_{a}$, as the following proposition.

**Proposition** **2.** *The active probability of a BT is given by the following:*

(10) $$\begin{array}{c} P_{a}=\frac{\alpha }{2\pi }\int_{0}^{\infty }\frac{\sin\left(\gamma \sin\frac{2\pi }{\alpha }\right){\left(\frac{\gamma }{W}\right)}^{\frac{\alpha }{2}}}{\gamma \exp\left({\frac{\gamma }{W}}^{\frac{\alpha }{2}}Q+\gamma \cos\left(\frac{2\pi }{\alpha }\right)\right)}d\gamma , \end{array}$$

*where $Q=\frac{1}{1-\eta }\left(c_{2}-\frac{1}{c_{1}}\ln\left(\frac{E_{\max}\left(1+\exp\left(c_{1}c_{2}\right)\right)}{E_{\max}+P_{c}\exp\left(c_{1}c_{2}\right)}-1\right)\right)$, and W is a function of both $G_{tm}$ and $\theta_{tm}$ given by Equation (9).*

**Proof.**  The proof is provided in Appendix C. □

**Remark** **1.** *The active probability given by Equation (10) is general for BTs that are equipped with directional antennas or omni-directional antennas. When we set $G_{tm}=1$ and $\theta_{tm}=2\pi$, we can have the active probability of a BT equipped with an omni-directional antenna. We can observe from Equation (10) that the active probability is a function of the gain of the main lobe of BTs ($G_{tm}$), the beamwidth of the main lobe of BTs ($\theta_{tm}$), the density of CEs ($\lambda_{CE}$), and the path loss exponent (α).*

### 3.2. Connectivity

For a BT-BR transmission pair, we consider that the signal transmitted from a BT can be received successfully when the signal-to-interference-noise-ratio (SINR) at the BR is not less than a threshold δ. Given the distance between the BT-BR pair by $R_{0}$, the connectivity of the BT-BR pair is provided by the following:

(11) $$\begin{array}{cc} P_{c} & =P(\mathrm{SIN}R\geq \delta ,P_{\mathrm{BT}}^{r}\geq Q)\\ \; & =P\left(\frac{\eta P_{\mathrm{BT}}^{r}R_{0}^{-\alpha }h_{0}G_{\mathrm{tm}}G_{\mathrm{rm}}}{I+\sigma^{2}}\geq \delta ,P_{\mathrm{BT}}^{r}\geq Q\right)\\ \; & =P\left(h_{0}\geq \frac{\delta (I+\sigma^{2})}{\eta P_{\mathrm{BT}}^{r}R_{0}^{-\alpha }G_{\mathrm{tm}}G_{\mathrm{rm}}},P_{\mathrm{BT}}^{r}\geq Q\right)\\ \; & =\int_{Q}^{\infty }E_{I}\left[e^{-\frac{\delta (I+\sigma^{2})}{\eta P_{\mathrm{BT}}^{r}R_{0}^{-\alpha }G_{\mathrm{tm}}G_{\mathrm{rm}}}}\right]\cdot f_{P_{\mathrm{BT}}^{r}}\left(x\right)dx\\ \; & =\int_{Q}^{\infty }L_{I}\left(B\right)\cdot e^{-B\sigma^{2}}\cdot f_{P_{\mathrm{BT}}^{r}}\left(x\right)dx, \end{array}$$

where $B=\delta /(\eta P_{\mathrm{BT}}^{r}R_{0}^{-\alpha }G_{\mathrm{tm}}G_{\mathrm{rm}})$, $h_{0}$ is the small-scale fading factor for the path between the studied BT and the studied BR, and $\sigma^{2}$ is the power of noise.

**Figure 6:**
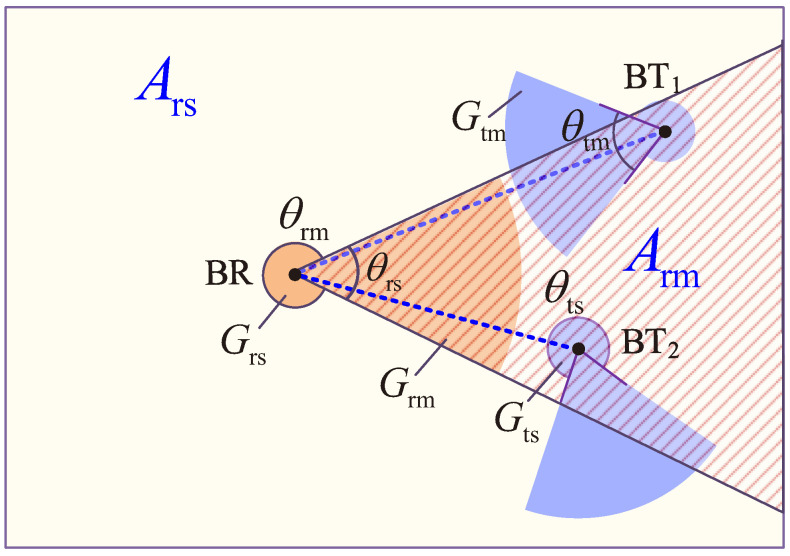
Example for illustrating the interference reception at a BR.

Next, we derive the Laplace transforms $L_{I}\left(B\right)$. We first analyze the interference reception at a BR (i.e., *I*). Figure 6 provides an example to illustrate the interference received at a BR. Because the directional antenna of a BR receives interference by two antenna gains (i.e., the gain of the main lobe and the gain of the side lobe), we divide the surrounding region of the BR into two regions:

- The region where a BR receives interference by the main lobe within $\theta_{\mathrm{rm}}$ (the shadowed region in Figure 6), named region $A_{\mathrm{rm}}$;
- The region where a BR receives interference by the side lobe within $\theta_{\mathrm{rm}}$, named region $A_{\mathrm{rs}}$.

Let $I_{A_{\mathrm{rm}}}$ and $I_{A_{\mathrm{rs}}}$ denote the received interference in regions $A_{\mathrm{rm}}$ and $A_{\mathrm{rs}}$, respectively. The received interference at a BR can be given by $I=I_{A_{\mathrm{rm}}}+I_{A_{\mathrm{rs}}}$.

We first analyze the received interference from region $A_{\mathrm{rm}}$ (i.e., $I_{A_{\mathrm{rm}}}$). The received interference in region $A_{\mathrm{rm}}$ can be split into two parts: (1) the interference transmitted by the main lobe of BTs (such as the BT_1_ in Figure 6), where the density of this type of BTs (pointing the BR by their main lobe) is $\theta_{\mathrm{tm}}/\left(2\pi \right)\cdot \left(\lambda_{\mathrm{BT}}/N\right)$; (2) the interference transmitted by the side lobe of BTs (such as BT_2_ in Figure 6), where the density of this type of BTs (pointing the BR by their side lobe) is $\theta_{\mathrm{ts}}/\left(2\pi \right)\cdot \left(\lambda_{\mathrm{BT}}/N\right)$. Because the orientation of directional antennas of BTs is mutually independently distributed, the BTs pointing a BR by their main lobes and the BTs pointing a BR by their side lobes are two independent HPPP distributions, denoted by $\Theta_{\mathrm{BT}}\left(A_{\mathrm{rm}}^{m}\right)$ and $\Theta_{\mathrm{BT}}\left(A_{\mathrm{rm}}^{s}\right)$, respectively. Let BT_0_ denote the BT paired with the BR being studied. Then, the received interference at the studied BR in region $A_{\mathrm{rm}}$ can be expressed as follows:

(12) $$\begin{array}{c} I_{A_{\mathrm{rm}}}=\sum_{{\mathrm{BT}}_{j}\in \Theta_{\mathrm{BT}}\left(A_{\mathrm{rm}}^{m}\right)\setminus {\mathrm{BT}}_{0}}P_{a}\eta P_{\mathrm{BT}}^{r}r_{j}^{-\alpha }h_{j}G_{\mathrm{rm}}G_{\mathrm{tm}}+\sum_{{\mathrm{BT}}_{j}\in \Theta_{\mathrm{BT}}\left(A_{\mathrm{rm}}^{s}\right)}P_{a}\eta P_{\mathrm{BT}}^{r}r_{j}^{-\alpha }h_{j}G_{\mathrm{rm}}G_{\mathrm{ts}}, \end{array}$$

where $r_{j}$ is the distance between BT_*j*_ and the studied BR, and $h_{j}$ is the small-scale fading factor for the path between BT_*j*_ and the studied BR.

Similarly, the received interference at the BR from region $A_{\mathrm{rs}}$ can be expressed as follows:

(13) $$\begin{array}{c} I_{A_{\mathrm{rs}}}=\sum_{{\mathrm{BT}}_{j}\in \Theta_{\mathrm{BT}}\left(A_{\mathrm{rs}}^{m}\right)}P_{a}\eta P_{\mathrm{BT}}^{r}r_{j}^{-\alpha }h_{j}G_{\mathrm{rs}}G_{\mathrm{tm}}+\sum_{{\mathrm{BT}}_{j}\in \Theta_{\mathrm{BT}}\left(A_{\mathrm{rs}}^{s}\right)}P_{a}\eta P_{\mathrm{BT}}^{r}r_{j}^{-\alpha }h_{j}G_{\mathrm{rs}}G_{\mathrm{ts}}, \end{array}$$

where $\Theta_{\mathrm{BT}}\left(A_{\mathrm{rs}}^{m}\right)$ and $\Theta_{\mathrm{BT}}\left(A_{\mathrm{rs}}^{s}\right)$ are HPPPs that BTs pointing the BR in region $A_{\mathrm{rs}}$ by their main lobes and side lobes, respectively. The two HPPPs $\Theta_{\mathrm{BT}}\left(A_{\mathrm{rs}}^{m}\right)$ and $\Theta_{\mathrm{BT}}\left(A_{\mathrm{rs}}^{s}\right)$ are independent, and $G_{\mathrm{rs}}=\frac{2-G_{\mathrm{rm}}\left(1-\cos\left(\frac{\theta_{\mathrm{rm}}}{2}\right)\right)}{\cos\frac{\theta_{\mathrm{rm}}}{2}-1}$.

With the received interference $I=I_{A_{\mathrm{rm}}}+I_{A_{\mathrm{rs}}}$, we then provide the Laplace transform $L_{I}\left(B\right)$ as the following Lemma.

**Lemma** **1.** *The Laplace transform $L_{I}\left(B\right)$ can be expressed as follows:*

(14) $$\begin{array}{cc} L_{I}\left(B\right)= & \exp\left(-\frac{\theta_{tm}\theta_{rm}\lambda_{BT}}{2\pi N}\int_{0}^{\infty }\left(1-\Phi_{1}\right)r_{j}dr_{j}\right)\times \\ \; & \exp\left(-\frac{\theta_{ts}\theta_{rm}\lambda_{BT}}{2\pi N}\int_{0}^{\infty }\left(1-\Phi_{2}\right)r_{j}dr_{j}\right)\times \\ \; & \exp\left(-\frac{\theta_{tm}\theta_{rs}\lambda_{BT}}{2\pi N}\int_{0}^{\infty }\left(1-\Phi_{3}\right)r_{j}dr_{j}\right)\times \\ & \exp\left(-\frac{\theta_{ts}\theta_{rs}\lambda_{BT}}{2\pi N}\int_{0}^{\infty }\left(1-\Phi_{4}\right)r_{j}dr_{j}\right), \end{array}$$

*where $\Phi_{1}\;\;=\;\;\int_{0}^{\infty }\;\;\exp\left(\;-\xi \theta_{tm}{\left(G_{tm}G_{tm}G_{rm}\right)}^{\frac{2}{\alpha }}\;\right)e^{-h}dh\cdot \;\;\int_{0}^{\infty }\exp\left(\;-\xi \theta_{ts}{\left(G_{ts}G_{tm}G_{rm}\right)}^{\frac{2}{\alpha }}\right)e^{-h}dh$,*

*$\Phi_{2}=\int_{0}^{\infty }\exp\left(-\xi \theta_{tm}{\left(G_{tm}G_{ts}G_{rm}\right)}^{\frac{2}{\alpha }}\right)e^{-h}dh\cdot \int_{0}^{\infty }\exp\left(-\xi \theta_{ts}{\left(G_{ts}G_{ts}G_{rm}\right)}^{\frac{2}{\alpha }}\right)e^{-h}dh$,*

*$\Phi_{3}=\int_{0}^{\infty }\exp\left(-\xi \theta_{tm}{\left(G_{tm}G_{tm}G_{rs}\right)}^{\frac{2}{\alpha }}\right)e^{-h}dh\cdot \int_{0}^{\infty }\exp\left(-\xi \theta_{ts}{\left(G_{ts}G_{tm}G_{rs}\right)}^{\frac{2}{\alpha }}\right)e^{-h}dh$,*

*$\Phi_{4}=\int_{0}^{\infty }\exp\left(-\xi \theta_{tm}{\left(G_{tm}G_{ts}G_{rs}\right)}^{\frac{2}{\alpha }}\right)e^{-h}dh\cdot \int_{0}^{\infty }\exp\left(-\xi \theta_{ts}{\left(G_{ts}G_{ts}G_{rs}\right)}^{\frac{2}{\alpha }}\right)e^{-h}dh$,*

*$\xi =\frac{\lambda_{CE}\pi {\left(BP_{a}\eta P_{CE}h\right)}^{\frac{2}{\alpha }}r_{j}^{-2}}{N\alpha \sin(\frac{2\pi }{\alpha })}$, and $P_{a}$ is given by Proposition 2.*

**Proof.**  The proof is provided in Appendix D. □

Finally, the connectivity of large-scale Dir-BackCom Nets can be given by the following Theorem.

**Theorem** **1.** *The connectivity of large-scale Dir-BackCom Nets can be expressed as follows:*

(15) $$\begin{array}{c} P_{c}\;\;=\;\;\int_{Q}^{\infty }\;\;\exp\left(\;\;-B\sigma^{2}\;\;-\;\frac{\lambda_{BT}}{2\pi N}\left(\theta_{tm}\theta_{rm}M_{1}+\theta_{ts}\theta_{rm}M_{2}+\;\theta_{tm}\theta_{rs}M_{3}+\theta_{ts}\theta_{rs}M_{4}\right)\;\;\right)f_{P_{BT}^{r}}\left(x\right)dx, \end{array}$$

*where $M_{n}=\int_{0}^{\infty }\left(1-\Phi_{n}\right)r_{j}dr_{j}$ for n={1,2,3,4}. The term $\Phi_{n}$(n=1,2,3,4) is given by Lemma 1. The term $f_{P_{BT}^{r}}\left(x\right)=\frac{\alpha }{2\pi }\int_{0}^{\infty }\frac{\sin\left(\gamma \sin\frac{2\pi }{\alpha }\right){\left(\frac{\gamma }{W}\right)}^{\frac{\alpha }{2}}}{\gamma \exp\left({\left(\frac{\gamma }{W}\right)}^{\frac{\alpha }{2}}x+\gamma \cos\frac{2\pi }{\alpha }\right)}d\gamma$ is given by Proposition 1.*

**Proof.**  After inserting both Equations (14) and (9) into Equation (11), we can obtain the connectivity given by Equation (15). □

**Remark** **2.** *The connectivity given by Equation (15) is general for large-scale BackCom Nets equipped with omn-directional antennas or directional antennas. Specifically, when $G_{tm}=G_{rm}=1$ and $\theta_{tm}=\theta_{rm}=2\pi$, we can have the connectivity of large-scale Omn-BackCom Nets. We can observe from Equation (15) that the connectivity is a function of the density of BTs ($\lambda_{BT}$), the antenna gain of main lobe of BTs and BRs ($G_{tm}$ and $G_{rm}$), the beamwidth of the main lobe of BTs and BRs ($\theta_{tm}$ and $\theta_{rm}$), and path loss exponent α. The specific relationship will be investigated in Section 5.*

## 4. Network Throughput

Considering our BackCom Net is a large-scale network, we adopt the spatial throughput as the metric of the network throughput [18,35]. The spatial throughput of BackCom Nets is defined as follows.

**Definition** **2.** *The spatial throughput of BackCom Nets is the throughput of BTs within a unit area of the network space.*

The spatial throughput of large-scale Dir-BackCom Nets can be expressed by the following.

(16) $$\begin{array}{cc} T & =\lambda_{\mathrm{BT}}E\left[\log_{2}\left(1+\mathrm{SIN}R\right)\right]. \end{array}$$

**Theorem** **2.** *The spatial throughput of large-scale Dir-BackCom Nets is given by the following:*

(17) $$\begin{array}{c} T\;=\;\lambda_{BT}\;\int_{\log_{2}\left(1+\delta \right)}^{\infty }\;\;\left(\int_{Q}^{\infty }\;\exp\left(-B\sigma^{2}\;-\;\frac{\lambda_{BT}}{2\pi }\left(\theta_{tm}\theta_{rm}M_{1}\;\;+\;\theta_{ts}\theta_{rm}M_{2}\;\;+\;\theta_{tm}\theta_{rs}M_{3}\;\;+\;\theta_{ts}\theta_{rs}M_{4}\;\right)\;\;\right)f_{P_{BT}^{r}}\left(x\right)dx\;\;\right)dt, \end{array}$$

*where $M_{n}=\int_{0}^{\infty }\left(1-\Phi_{n}\right)r_{j}dr_{j}$ for n={1,2,3,4}, and $\Phi_{n}$ is given by Lemma 1. The term $f_{P_{BT}^{r}}\left(x\right)$ is given by Proposition 1. Note that term B in Equation (17) needs to be replaced by $B=\left(e^{t}-1\right)/\left(\eta P_{BT}^{r}R_{0}^{-\alpha }G_{tm}G_{rm}\right)$.*

**Proof.**  The proof is provided in Appendix E. □

## 5. Simulations and Numerical Results

We conduct simulations to verify our proposed analytical model. Specifically, we adopt commercial software MATLAB as our simulation tool. In simulations, fixed parameters/factors are provided in Table 1. Then, simulations are conducted in two groups: In Group 1, we make a comparison of performance among different BackCom Nets, such as Dir-BackCom Nets, Dir-Omn-BackCom Nets, and Omn-BackCom Nets; in Group 2, we make a comparison of performance among Dir-BackCom Nets with different directional antennas having various parameters. Our simulation results are obtained by the Monte Carlo simulation averaged by 3000 topological trials. In the following figures, the abbreviations ’ana’ and ’sim’ in legends stand for ’analysis’ and ’simulation’, respectively.

### 5.1. Comparison among Dir-BackCom Nets, Dir-Omn-BackCom Nets, and Omn-BackCom Nets

**Figure 7:**
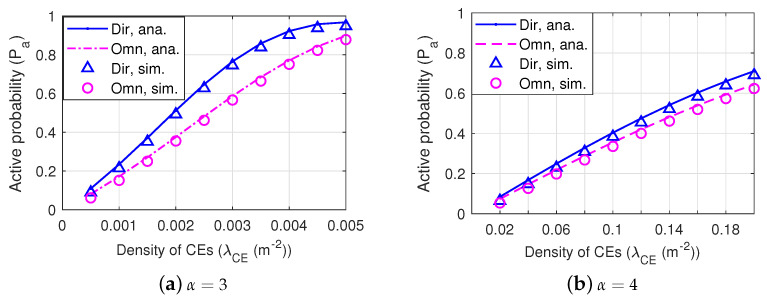
The active probability of a BT equipped with an omni-directional antenna or a directional antenna with parameters $\theta_{\mathrm{tm}}=1.75$ rad and $G_{\mathrm{tm}}=6$.

Table 2 gives the difference among Dir-BackCom Nets, Dir-Omn-BackCom Nets, and Omn-BackCom Nets. We can see in Table 2 that in both Dir-BackCom Nets and Dir-Omn-BackCom Nets, BTs are equipped with directional antennas, while in Omn-BackCom Nets, BTs are equipped with omni-directional antennas. Then, we first compare the active probability in the network in which BTs are equipped with a directional antenna or an omni-directional antenna. Figure 7 shows the active probability of a BT versus the density of CEs under different path loss exponents in the network in which BTs have different antennas. We can observe that the active probability of a BT with a directional antenna is always higher than that with an omni-directional antenna under different network environments (i.e., α=3 and α=4). This phenomenon indicates that compared with the omni-directional reception, directional antennas can receive more RF signals by beamforming reception with a higher gain, implying that employing directional antennas at BTs can improve the active probability. In addition, we can observe that the active probability increases with the increment of the density of CEs and decreases with the increment of the path loss exponent. This is because BTs can receive more RF signals when there are more CEs or when the path loss is low in a network environment.

**Figure 8:**
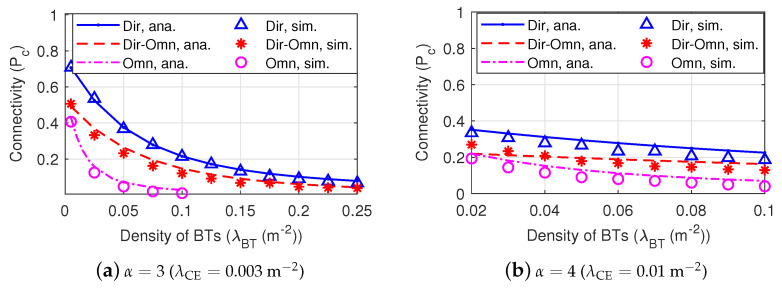
The connectivity among Dir-BackCom Nets, Omn-Dir-BackCom, and Omn-BackCom Nets, where $R_{0}=3$ m. The parameters of directional antennas: $\theta_{\mathrm{tm}}=1.75$ rad and $G_{\mathrm{tm}}=6$.

Figure 8 shows the connectivity among Dir-BackCom Nets, Dir-Omn BackCom, and Omn-BackCom Nets under different density of BTs and various path loss exponents. We can observe that the connectivity of *Dir-Omn-BackCom Nets* is always higher than *Omn-BackCom Nets* under different path loss environments (i.e., α = 3 and α = 4). From Figure 7, we know that BTs with directional antennas can have a higher active probability than BTs with omni-directional antennas. Then, the higher active probability of BTs with directional antennas can increase the quantity of transmitting BTs, thereby increasing the overall interference of the network. However, Figure 8 shows that compared with omni-directional antennas, using directional antennas at BTs can still improve the network connectivity, although with higher interferences. The reasons of this phenomenon are as follows: (1) BTs with directional antennas can have higher active probabilities; (2) the beamforming with a higher gain also can strengthen transmitted signals, thereby leading to a higher SINR. In addition, we can observe in Figure 8 that the connectivity of *Dir-BackCom Nets* is higher than *Dir-Omn-BackCom Nets* under different values of α, indicating that using directional antenna at BRs can also improve the network’s connectivity. This is because the narrower beamforming with a higher gain of directional antennas can receive a higher SINR by both augmenting the signal strength in the intended direction and reducing the interference in undesired directions. Therefore, we can conclude that using directional antennas at BTs or BRs can both improve the connectivity of BackCom Nets. In addition, we can observe that the connectivity decreases with the increment of the density of BTs. This is because more BTs lead to higher interferences in BackCom Nets.

**Figure 9:**
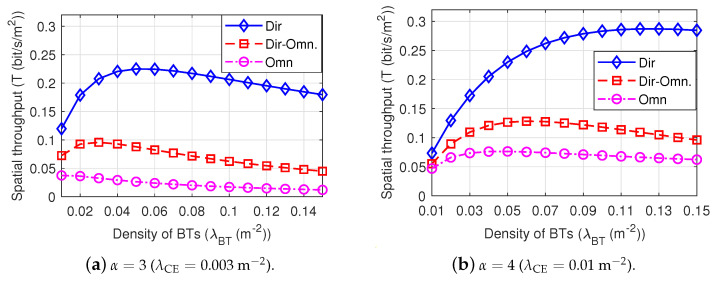
The spatial throughput among Dir-BackCom Nets, Omn-Dir-BackCom, and Dir-BackCom Nets, where $R_{0}=3$ m. The parameters of directional antennas: $\theta_{\mathrm{tm}}=1.75$ rad and $G_{\mathrm{tm}}=6$.

Figure 9 shows the spatial throughput among Dir-BackCom Nets, Dir-Omn BackCom, and Omn-BackCom Nets under different path loss exponents. We can observe that the spatial throughput of Dir-BackCom Nets is significantly higher than both Omn-Dir-BackCom and Dir-BackCom Nets, indicating that equipping directional antennas at BTs and BRs instead of omni-directional antennas can greatly improve the spatial throughput of BackCom Nets. Moreover, we can observe in Figure 9 that there is an optimal density of BTs to maximize the spatial throughput. The optimal density of BTs of Dir-BackCom Nets is higher than both Dir-Omn-BackCom Nets and Omn-BackCom Nets, indicating that Dir-BackCom Nets can accommodate more BTs to obtain a maximum throughput in a unit area compared with Dir-Omn-BackCom Nets and Omn-BackCom Nets. This phenomenon also indicates that we can choose a proper density of BTs to improve the spatial throughput according to different BackCom Nets.

### 5.2. Comparison among Dir-BackCom Nets with Different Directional Antennas

In this group, we investigate the impact of different directional antennas on the performance of Dir-BackCom Nets. Specifically, we consider that these different directional antennas possess different beamwidths. Meanwhile, similarly to [38], the considered directional antennas with a narrower beamwidth of the main lobe have a higher gain of the main lobe. Then, we consider that for different Dir-BackCom Net, directional antennas of BTs have the following varied parameters: $\left(\theta_{\mathrm{tm}},G_{\mathrm{tm}}\right)=\left\{\left(2.13\;\mathrm{rad},\;4\right),\left(1.75\;\mathrm{rad},\;6\right),\left(1.35\;\mathrm{rad},10\right)\right\}$. In addition, the antenna parameters of BRs are fixed as $\left(\theta_{\mathrm{rm}},\;G_{\mathrm{rm}}\right)=\left(1.75\;\mathrm{rad},\;6\right)$. It is worth noting that we do not investigate the impact of antenna beamwidth of BRs on performance, because BRs have a narrower beamwidth and the higher gain of the main lobe can obviously lead to a better network performance due to its high signal strength and lower interference.

**Figure 10:**
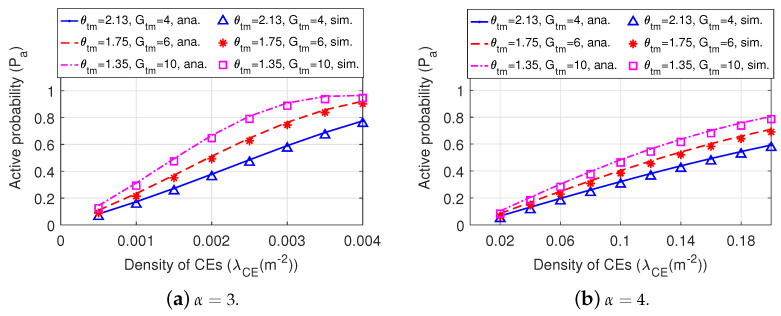
The active probability of a BT in Dir-BackCom Nets. In different Dir-BackCom Nets, directional antennas of BTs have different values with respect to antenna beamwidths and gains of the main lobe.

Figure 10 shows the active probability of a BT versus the density of CEs in the network with different values of antenna beamwidth and antenna gain of the main lobe. We can observe that directional antennas of BTs possessing a narrower beamwidth and a higher gain of the main lobe can acquire higher active probability under different path loss environments (i.e., α=3 and α=4), indicating that directional antennas possessing narrower beamforming with a higher gain of the main lobe can improve the active probability.

**Figure 11:**
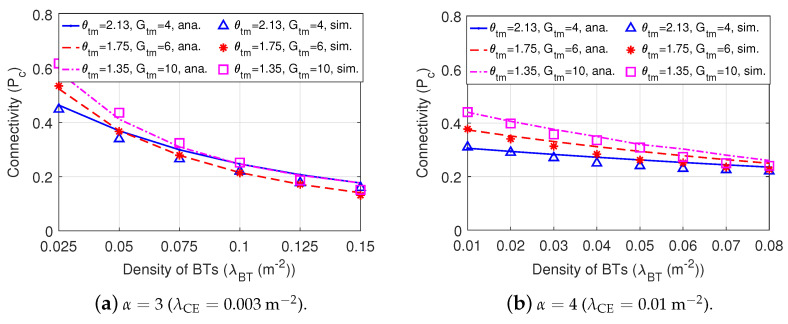
The connectivity of Dir-BackCom Nets. In different Dir-BackCom Nets, directional antennas of BTs have different values of antenna beamwidth and gain of the main lobe: parameter $R_{0}=3$ m.

Figure 11 shows the connectivity of Dir-BackCom versus the density of BTs when the directional antennas of BTs in different Dir-BackCom Nets have different values of antenna beamwidth and gain of the main lobe. In Figure 11, we can observe that the connectivity varies in various antenna beamwidth and gain. Specifically, when α=3 ($\lambda_{\mathrm{CE}}=0.003\;$$m^{-2}$), the connectivity increases with the narrower beamwidth and higher gain under 0.025$m^{-2}\leq \lambda_{\mathrm{BT}}\leq 0.1\;$$m^{-2}$; when α=4 ($\lambda_{\mathrm{CE}}=0.01\;$$m^{-2}$), the connectivity increases with the narrower beamwidth and higher gain under 0.01 $m^{-2}\leq \lambda_{\mathrm{BT}}\leq 0.08\;$$m^{-2}$. This phenomenon implies that we can choose a proper antenna beamwidth and gain of the main lobe for the directional antennas of BTs to improve the connectivity of BackCom Nets.

**Figure 12:**
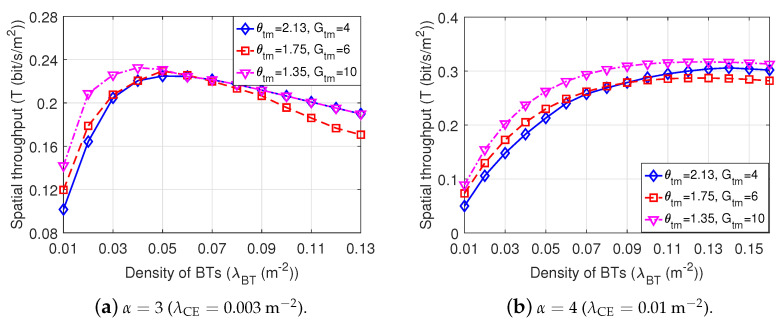
The Spatial throughput of Dir-BackCom Nets. In different Dir-BackCom Nets, directional antennas of BTs have different values of antenna beamwidth and gain of the main lobe: parameter $R_{0}=3$ m.

Figure 12 shows the spatial throughput of Dir-BackCom Nets, where the directional antennas of BTs in different Dir-BackCom Nets have different values of antenna beamwidths and gains of the main lobe. We can observe in Figure 12 that Dir-BackCom Nets equipped with directional antennas possessing the narrowest beamwidth and the highest gain of the main lobe can obtain the highest spatial throughput when 0.01 $m^{-2}$$\leq \lambda_{\mathrm{BT}}\leq 0.13\;$$m^{-2}$ under $\alpha =3\;(\lambda_{\mathrm{CE}}=0.003\;$$m^{-2}$) and when 0.01$m^{-2}$$\leq \lambda_{\mathrm{BT}}\leq 0.15\;$$m^{-2}$ under $\alpha =4\;(\lambda_{\mathrm{CE}}=0.01\;$$m^{-2}$). This phenomenon indicates that the spatial throughput can be improved by choosing a directional antenna with a proper antenna beamwith and gain. In addition, for each directional antenna with specific beamwidth and gain of the main lobe, there is an optimal density of BTs to maximize the spatial throughput, indicating that we can choose an optimal density to improve the spatial throughput of Dir-BackCom based on different directional antennas.

## 6. Conclusions

This article establishes a theoretic model to analyze the performance of large-scale Dir-BackCom Nets, where both BTs and BRs are equipped with directional antennas. The performance metrics include both connectivity and spatial throughput. Our theoretic model is general for BackCom Nets where BTs/BRs are equipped with directional antennas or omni-directional antennas. The accuracy of our theoretic model is verified by extensive simulations. In conclusion, this paper provides the following major findings:

- Equipping directional antennas instead of omni-directional antenna at BTs can improve the active probability.
- Employing directional antennas at either BTs or BRs can improve the connectivity and spatial throughput of BackCom Nets.
- The spatial throughput can be maximized by choosing an optimal density of BTs.
- Both the connectivity and spatial throughput of BackCom Nets can be improved by choosing a directional antenna with a proper antenna beamwidth and gain of the main lobe.

## Figures and Tables

**Table 1:** Parameter/factor setting.

Parameters	Values
The number of available frequency channels (*N*)	5
The transmitted power of CEs ($P_{\mathrm{CE}}$)	19.5 dBm [7]
The reflection coefficient (η)	0.375 [7,8]
The power of noise ($\sigma^{2}$)	−60 dBm [7,8]
The threshold of SINR (δ)	5 dB [7]
The power threshold for activating the circuits	
of backscatter communications ($P_{c}$)	10.6μW [36]
The saturated energy power at the energy harvester ($E_{\max}$)	48.86μW [37]
The factors in the energy harvester $\left(c_{1},c_{2}\right)$	26515.46, −0.00002981 [37]

**Table 2:** Antenna types in different BackCom Nets.

	Dir-BackCom Nets	Dir-Omn-BackCom Nets	Omn-BackCom Nets
**BTs**	Directional antennas	Directional antennas	Omni-directional antennas
**BRs**	Directional antennas	Omni-directional antennas	Omni-directional antennas

## Data Availability

Not applicable.

## Abbreviations

The following abbreviations are used in this manuscript:

BackCom	Backscatter communication;
BackCom Net	Backscatter communication network;
RF	Radio frequency;
IoT	Internet of things;
BT	Backscatter transmitter;
CE	Carrier emitter;
BR	Backscatter receiver;
WPT	Wireless power transfer;
RFID	Radio frequency identification;
HPPP	Homogeneous Poisson point process;
UCA	Uniform circular array;
HPBW	Half-power beamwidth;
CDF	Cumulative distribution function;
PDF	Probability density function;
SINR	Signal-to-interference-noise-ratio;
PGFL	Probability generating functional;

## Appendix A. Deviation of the Antenna Gain of the Keyhole Antenna Model

The radiation power of the keyhole antenna model, denoted by $P_{rad}$, consists of the main lobe power denoted by $P_{m}$ and the side lobe power denoted by $P_{s}$, i.e., the radiation power of the keyhole antenna model can be given the following:

(A1) $$P_{rad}=P_{s}+P_{m},$$

where $P_{rad}=4\pi U_{0}$, and $U_{0}$ is the constant radiation strength for a reference isotropic antenna in a solid angle (θ,ϕ). The terms $P_{m}$ and $P_{s}$ can be expressed by the following two equations, respectively.

(A2) $$P_{m}=\int_{0}^{2\pi }\int_{0}^{\frac{\theta_{m}}{2}}G_{m}U_{0}\sin\theta d\theta d\phi ,$$

(A3) $$P_{s}=\int_{0}^{2\pi }\int_{\frac{\theta_{m}}{2}}^{2\pi }G_{s}U_{0}\sin\theta d\theta d\phi .$$

After combining Equations (A1)–(A3), the gain of the side lobe of the keyhole antenna model, denoted by $G_{s}$, can be given by the following.

(A4) $$G_{s}=\frac{2-G_{m}\left(1-\cos\left(\frac{\theta_{m}}{2}\right)\right)}{\cos\frac{\theta_{m}}{2}-1}.$$

After projecting the three-dimension antenna gain to two dimensions, we can have the two-dimension antenna gain of the keyhole antenna model provided in Equation (1).

## Appendix B. Proof of Proposition 1

We recall two assumptions of our channel model: (1) the density of co-channel CEs is $\lambda_{\mathrm{CE}}/N$; (2) a BT only can choose one channel to receive RF signals, and we can see that the density of CEs that provide RF signals for a BT is $\lambda_{\mathrm{CE}}/N$. Thus, the density of both HPPPs $\Theta_{\mathrm{CE}}\left(A_{\mathrm{tm}}\right)$ (CEs providing power in region $A_{\mathrm{tm}}$) and $\Theta_{\mathrm{CE}}\left(A_{\mathrm{ts}}\right)$ (CEs providing power in region $A_{\mathrm{ts}}$) is $\lambda_{\mathrm{CE}}/N$.

Then, the CDF of the received power at a BT ($P_{\mathrm{BT}}^{r}$) can be derived by utilizing a Laplace transform and inverse Laplace transform. The Laplace transform of $P_{\mathrm{BT}}^{r}$ can be expressed as follows:

(A5) $$L_{P_{\mathrm{BT}}^{r}}\left(s\right)\;=\;E\left[e^{-sP_{\mathrm{BT}}^{r}}\;\right]\;\;=\;\;E\left[e^{-s\left(P_{\mathrm{BT}}^{A_{\mathrm{tm}}}+P_{\mathrm{BT}}^{A_{\mathrm{ts}}}\right)}\;\right]\;\;\overset{\left(a\right)}{=}\;\;E\left[e^{-sP_{\mathrm{BT}}^{A_{\mathrm{tm}}}}\;\right]\;\;\cdot \;E\left[e^{-sP_{\mathrm{BT}}^{A_{\mathrm{ts}}}}\;\right]\;\;=\;\;L_{P_{\mathrm{BT}}^{A_{\mathrm{tm}}}}\left(s\right)\;\cdot \;L_{P_{\mathrm{BT}}^{A_{\mathrm{ts}}}}\left(s\right),\;\;\;\;$$

where E[x] denotes the expectation of *x*, $L_{x}\left(s\right)$ is the Laplace transform and $L_{x}\left(s\right)=E\left[e^{-sx}\right]$. The process (a) results from the fact that the two HPPPs of CEs distributed in regions $A_{\mathrm{tm}}$ and $A_{\mathrm{ts}}$ are independent with each other. As a result, variables $P_{\mathrm{BT}}^{A_{\mathrm{tm}}}$ and $P_{\mathrm{BT}}^{A_{\mathrm{ts}}}$ are independent. The Laplace transform $L_{P_{\mathrm{BT}}^{A_{\mathrm{tm}}}}\left(s\right)$ can be given by the following:

(A6) $$\begin{array}{cc} L_{P_{\mathrm{BT}}^{A_{\mathrm{tm}}}}\left(s\right) & =E\left[e^{-s\;\;\sum_{{\mathrm{CE}}_{i}\in \Theta_{\mathrm{CE}}^{A_{\mathrm{tm}}}}P_{\mathrm{CE}}r_{i}^{-\alpha }h_{i}G_{\mathrm{tm}}}\right]\\ \; & =E_{r_{i},h_{i}}\left[\prod_{{\mathrm{CE}}_{i}\in \Theta_{\mathrm{CE}}^{A_{\mathrm{tm}}}}e^{-sP_{\mathrm{CE}}r_{i}^{-\alpha }h_{i}G_{\mathrm{tm}}}\right]\\ \; & \overset{\left(b\right)}{=}E_{r_{i}}\prod_{{\mathrm{CE}}_{i}\in \Theta_{\mathrm{CE}}^{A_{\mathrm{tm}}}}E_{h_{i}}\left[e^{-sP_{\mathrm{CE}}r_{i}^{-\alpha }h_{i}G_{\mathrm{tm}}}\right]\\ \; & \overset{\left(c\right)}{=}\exp\left(-\frac{\lambda_{\mathrm{CE}}}{N}\int_{0}^{\theta_{\mathrm{tm}}}\int_{0}^{\infty }\left(1-E_{h_{i}}\left[sP_{\mathrm{CE}}r_{i}^{-\alpha }h_{i}G_{\mathrm{tm}}\right]\right)r_{i}dr_{i}d\theta_{i}\right)\\ \; & =\exp\left(-\frac{\lambda_{\mathrm{CE}}}{N}\int_{0}^{\theta_{\mathrm{tm}}}\int_{0}^{\infty }\left(1-\frac{1}{1+sP_{\mathrm{CE}}r_{i}^{-\alpha }G_{\mathrm{tm}}}\right)r_{i}dr_{i}d\theta_{i}\right)\\ \; & =\exp\left(-\frac{\lambda_{\mathrm{CE}}}{N}\cdot \frac{\pi \theta_{\mathrm{tm}}{\left(sP_{\mathrm{CE}}G_{\mathrm{tm}}\right)}^{\frac{2}{\alpha }}}{\alpha \sin(\frac{2\pi }{\alpha })}\right)\\ \; & =\exp\left(-W_{1}s^{\frac{2}{\alpha }}\right), \end{array}$$

where $W_{1}\;\;=\;\;\frac{\pi \lambda_{\mathrm{CE}}\theta_{\mathrm{tm}}{\left(P_{\mathrm{CE}}G_{\mathrm{tm}}\right)}^{\frac{2}{\alpha }}}{N\alpha \sin(\frac{2\pi }{\alpha })}$, (b) results from the fact that variables $r_{i}$ and $h_{i}$ are independent with each other. Process (c) is obtained by using a property of a PPP as follows: for a PPP Θ in space *S*, if *r* and θ denote the polar radius and the polar angle of points in the PPP, respectively, the probability generating functional (PGFL) of the PPP can be expressed as $E\left[\prod_{r\in \Theta }v\left(r\right)\right]=\exp\left(\;-\lambda \;\;{\;\int \int \;}_{S}\left(\;1-v\left(r\right)\right)rdrd\theta \right)$, where v(r) is a function of *r* [7,39].

After employing a similar calculation with Equation (A6) and simultaneously replacing terms ${\mathrm{CE}}_{i}\in \Theta_{\mathrm{CE}}^{A_{\mathrm{tm}}}$, $G_{\mathrm{tm}}$, and $\theta_{\mathrm{tm}}$ by terms ${\mathrm{CE}}_{i}\in \Theta_{\mathrm{CE}}^{A_{\mathrm{ts}}}$, $G_{\mathrm{ts}}$, and $\theta_{\mathrm{ts}}$, respectively, the Laplace transform $L_{P_{\mathrm{BT}}^{A_{\mathrm{ts}}}}\left(s\right)$ in Equation (A5) can be given by the following:

(A7) $$\begin{array}{c} L_{P_{\mathrm{BT}}^{A_{\mathrm{ts}}}}\left(s\right)=\exp\left(-W_{2}s^{\frac{2}{\alpha }}\right), \end{array}$$

where $W_{2}=\frac{\pi \lambda_{\mathrm{CE}}\theta_{\mathrm{ts}}{\left(P_{\mathrm{CE}}G_{\mathrm{ts}}\right)}^{\frac{2}{\alpha }}}{N\alpha \sin(\frac{2\pi }{\alpha })}$.

Then, $L_{P_{\mathrm{BT}}^{r}}\left(s\right)$ can be given by the following:

(A8) $$\begin{array}{c} L_{P_{\mathrm{BT}}}\left(s\right)=\exp\left(-W_{1}s^{\frac{2}{\alpha }}\right)\exp\left(-W_{2}s^{\frac{2}{\alpha }}\right)=\exp\left(-Ws^{\frac{2}{\alpha }}\right), \end{array}$$

where $W=\frac{\pi \lambda_{\mathrm{CE}}P_{\mathrm{CE}}^{\frac{2}{\alpha }}\left(\theta_{\mathrm{tm}}G_{\mathrm{tm}}^{\frac{2}{\alpha }}+\theta_{\mathrm{ts}}G_{\mathrm{ts}}^{\frac{2}{\alpha }}\right)}{N\alpha \sin(\frac{2\pi }{\alpha })}$.

Next, the CDF of $P_{\mathrm{BT}}^{r}$ can be derived by employing the inverse Laplace transform on $L_{P_{\mathrm{BT}}^{r}}\left(s\right)$ as follows:

(A9) $$\begin{array}{cc} F_{P_{\mathrm{BT}}^{r}}\left(x\right)= & \frac{1}{2\pi j}\lim_{T\to \infty }\int_{c-jT}^{c+jT}\frac{\exp(sx-Ws^{\frac{2}{\alpha }})}{s}ds\\ \overset{\left(d\right)}{=} & 1-\frac{\alpha }{2\pi }\int_{0}^{\infty }\frac{\sin(\gamma \sin\frac{2\pi }{\alpha })}{\gamma \exp\left({\left(\frac{\gamma }{W}\right)}^{\frac{\alpha }{2}}x+\gamma \cos\frac{2\pi }{\alpha }\right)}d\gamma , \end{array}$$

where (d) can be obtained by applying Bromwich inversion with the modified contour [40,41].

After taking derivative of the CDF of $P_{\mathrm{BT}}^{r}$ given by Equation (A9), we can obtain the PDF of $P_{\mathrm{BT}}^{r}$ provided by Equation (9).

## Appendix C. Proof of Proposition 2

A BT can be activated if the backscatter condition: $P_{e}\left(P_{\mathrm{BT}}^{h}\right)\geq P_{c}$ is satisfied. By combining Equations (3)–(6), the backscatter condition can be transferred as follows.

(A10) $$\begin{array}{c} P_{\mathrm{BT}}^{r}\geq \frac{1}{1-\eta }\left(c_{2}-\frac{1}{c_{1}}\ln\left(\frac{E_{\max}\left(1+\exp\left(c_{1}c_{2}\right)\right)}{E_{\max}+P_{c}\exp\left(c_{1}c_{2}\right)}-1\right)\right). \end{array}$$

Let *Q* be equal to the right hand side of Equation (A10), i.e., $Q\;\;=\;\;\frac{1}{1-\eta }\left(\;c_{2}\;-\;\frac{1}{c_{1}}\;\ln\left(\;\frac{E_{\max}\left(1+\exp\left(c_{1}c_{2}\right)\right)}{E_{\max}+P_{c}\exp\left(c_{1}c_{2}\right)}\;\;-\;\;1\;\right)\;\right)$. Then, $P_{a}$ can be given by the following.

(A11) $$\begin{array}{c} P_{a}=P\left(P_{\mathrm{BT}}^{r}>Q\right)=1-F_{P_{\mathrm{BT}}^{r}}\left(Q\right). \end{array}$$

By inserting Equation (8) in Equation (A11), we can have $P_{a}$ provided in Equation (10).

## Appendix D. Proof of Lemma 1

The Laplace transform $L_{I}\left(B\right)$ can be expressed as follows:

(A12) $$\begin{array}{c} L_{I}\left(B\right)=E\left[e^{-B(I_{A_{\mathrm{rm}}}+I_{A_{\mathrm{rs}}})}\right]\overset{\left(e\right)}{=}E\left[e^{-BI_{A_{\mathrm{rm}}}}\right]\cdot E\left[e^{-BI_{A_{\mathrm{rs}}}}\right], \end{array}$$

where (e) results from the fact that the two HPPPs of BTs distributed in region $A_{\mathrm{rm}}$ and $A_{\mathrm{rs}}$ are independent with each other [34]. The terms $E\left[e^{-BI_{A_{\mathrm{rm}}}}\right]$ and $E\left[e^{-BI_{A_{\mathrm{rs}}}}\right]$ can be expressed as the following two equations:

(A13) $$\begin{array}{cc} E\left[e^{-BI_{A_{\mathrm{rm}}}}\right] & =E\left[e^{-B\left(\sum_{{\mathrm{BT}}_{j}\in \Theta_{\mathrm{BT}}\left(A_{\mathrm{rm}}^{m}\right)\setminus {\mathrm{BT}}_{0}}P_{a}\eta P_{\mathrm{BT}}^{r}r_{j}^{-\alpha }h_{j}G_{\mathrm{rm}}G_{\mathrm{tm}}+\sum_{{\mathrm{BT}}_{j}\in \Theta_{\mathrm{BT}}\left(A_{\mathrm{rm}}^{s}\right)}P_{a}\eta P_{\mathrm{BT}}^{r}r_{j}^{-\alpha }h_{j}G_{\mathrm{rm}}G_{\mathrm{ts}}\right)}\right]\\ \; & \overset{\left(f\right)}{=}E\left[e^{-B\sum_{{\mathrm{BT}}_{j}\in \Theta_{\mathrm{BT}}\left(A_{\mathrm{rm}}^{m}\right)\setminus {\mathrm{BT}}_{0}}P_{a}\eta P_{\mathrm{BT}}^{r}r_{j}^{-\alpha }h_{j}G_{\mathrm{rm}}G_{\mathrm{tm}}}\right]\cdot E\left[e^{-B\sum_{{\mathrm{BT}}_{j}\in \Theta_{\mathrm{BT}}\left(A_{\mathrm{rm}}^{s}\right)}P_{a}\eta P_{\mathrm{BT}}^{r}r_{j}^{-\alpha }h_{j}G_{\mathrm{rm}}G_{\mathrm{ts}}}\right]\\ \; & \overset{\left(g\right)}{=}\underset{\Sigma_{1}}{\underset{︸}{E\left[e^{-B\sum_{{\mathrm{BT}}_{j}\in \Theta_{\mathrm{BT}}\left(A_{\mathrm{rm}}^{m}\right)}P_{a}\eta P_{\mathrm{BT}}^{r}r_{j}^{-\alpha }h_{j}G_{\mathrm{rm}}G_{\mathrm{tm}}}\right]}}\cdot \underset{\Sigma_{2}}{\underset{︸}{E\left[e^{-B\sum_{{\mathrm{BT}}_{j}\in \Theta_{\mathrm{BT}}\left(A_{\mathrm{rm}}^{s}\right)}P_{a}\eta P_{\mathrm{BT}}^{r}r_{j}^{-\alpha }h_{j}G_{\mathrm{rm}}G_{\mathrm{ts}}}\right]}}, \end{array}$$

(A14) $$\begin{array}{cc} E\left[e^{-BI_{A_{\mathrm{rs}}}}\right] & =E\left[e^{-B\left(\sum_{{\mathrm{BT}}_{j}\in \Theta_{\mathrm{BT}}\left(A_{\mathrm{rs}}^{m}\right)}P_{a}\eta P_{\mathrm{BT}}^{r}r_{j}^{-\alpha }h_{j}G_{\mathrm{rs}}G_{\mathrm{tm}}+\sum_{{\mathrm{BT}}_{j}\in \Theta_{\mathrm{BT}}\left(A_{\mathrm{rs}}^{s}\right)}P_{a}\eta P_{\mathrm{BT}}^{r}r_{j}^{-\alpha }h_{j}G_{\mathrm{rs}}G_{\mathrm{ts}}\right)}\right]\\ \; & \overset{\left(h\right)}{=}\underset{\Sigma_{3}}{\underset{︸}{E\left[e^{-B\sum_{{\mathrm{BT}}_{j}\in \Theta_{\mathrm{BT}}\left(A_{\mathrm{rs}}^{m}\right)}P_{a}\eta P_{\mathrm{BT}}^{r}r_{j}^{-\alpha }h_{j}G_{\mathrm{rs}}G_{\mathrm{tm}}}\right]}}\cdot \underset{\Sigma_{4}}{\underset{︸}{E\left[e^{-B\sum_{{\mathrm{BT}}_{j}\in \Theta_{\mathrm{BT}}\left(A_{\mathrm{rs}}^{s}\right)}P_{a}\eta P_{\mathrm{BT}}^{r}r_{j}^{-\alpha }h_{j}G_{\mathrm{rs}}G_{\mathrm{ts}}}\right]}}, \end{array}$$

where (f) results from the fact that both point distribution processes ${\mathrm{BT}}_{j}\in \Theta_{\mathrm{BT}}\left(A_{\mathrm{rm}}^{m}\right)\setminus {\mathrm{BT}}_{0}$ and ${\mathrm{BT}}_{j}\in \Theta_{\mathrm{BT}}\left(A_{\mathrm{rm}}^{m}\right)$ are proven to be an identical point distribution process according to Slivnyak’s Theorem [42]. Process (g) is based on the fact that the two HPPPs, $\Theta_{\mathrm{BT}}\left(A_{\mathrm{rm}}^{m}\right)$ and $\Theta_{\mathrm{BT}}\left(A_{\mathrm{rm}}^{s}\right)$, are independent. Process (h) results from the fact that the two HPPPs, $\Theta_{\mathrm{BT}}\left(A_{\mathrm{rs}}^{m}\right)$ and $\Theta_{\mathrm{BT}}\left(A_{\mathrm{rs}}^{s}\right)$, are independent.

Next, we first derive $\Sigma_{1}$. Then, $\Sigma_{2}$, $\Sigma_{3}$, and $\Sigma_{4}$ can be derived by using the similar calculation process with $\Sigma_{1}$. Expression $\Sigma_{1}$ can be provided by the following:

(A15) $$\begin{array}{cc} \Sigma_{1} & =E\left[e^{-B\sum_{{\mathrm{BT}}_{j}\in \Theta_{\mathrm{BT}}\left(A_{\mathrm{rm}}^{m}\right)}P_{a}\eta P_{\mathrm{BT}}^{r}r_{j}^{-\alpha }h_{j}G_{\mathrm{rm}}G_{\mathrm{tm}}}\right]\\ \; & =E\left[\prod_{{\mathrm{BT}}_{j}\in \Theta_{\mathrm{BT}}\left(A_{\mathrm{rm}}^{m}\right)}e^{-BP_{a}\eta P_{\mathrm{BT}}^{r}r_{j}^{-\alpha }h_{j}G_{\mathrm{tm}}G_{\mathrm{rm}}}\right]\\ \; & \overset{\left(i\right)}{=}E_{r_{j}}\left[\prod_{{\mathrm{BT}}_{j}\in \Theta_{\mathrm{BT}}\left(A_{\mathrm{rm}}^{m}\right)}E\left[e^{-BP_{a}\eta P_{\mathrm{BT}}^{r}r_{j}^{-\alpha }h_{j}G_{\mathrm{tm}}G_{\mathrm{rm}}}\right]\right]\\ & \overset{\left(j\right)}{=}\exp\left(-\frac{\theta_{\mathrm{tm}}\lambda_{\mathrm{BT}}}{2\pi N}\int_{0}^{\theta_{\mathrm{rm}}}\int_{0}^{\infty }\left(1-E\left[e^{-BP_{a}\eta P_{\mathrm{BT}}^{r}r_{j}^{-\alpha }h_{j}G_{\mathrm{tm}}G_{\mathrm{rm}}}\right]\right)r_{j}dr_{j}d\theta_{j}\right)\\ \; & =\exp\left(-\frac{\theta_{\mathrm{tm}}\theta_{\mathrm{rm}}\lambda_{\mathrm{BT}}}{2\pi N}\int_{0}^{\infty }\left(1-\underset{\Phi_{1}}{\underset{︸}{E\left[e^{-BP_{a}\eta P_{\mathrm{BT}}^{r}r_{j}^{-\alpha }h_{j}G_{\mathrm{tm}}G_{\mathrm{rm}}}\right]}}\right)r_{j}dr_{j}\right), \end{array}$$

where (i) results from the fact that variable $r_{j}$ is independent with other variables (i.e., $h_{j}$ and $P_{\mathrm{BT}}$). The process (j) is obtained by employing the PGFL property of a PPP: $E\left[\prod_{r\in \Theta }v\left(r\right)\right]=\exp\left(-\lambda {\;\int \int \;}_{S}\left(1-v\left(r\right)\right)rdrd\theta \right)$.

After inserting the expression of $P_{\mathrm{BT}}^{r}$ given by Equation (7), expression $\Phi_{1}$ in Equation (A15) can be expressed as follows:

(A16) $$\begin{array}{cc} \Phi_{1} & =E\left[e^{-BP_{a}\eta \left(\sum_{{\mathrm{CE}}_{i}\in \Theta_{\mathrm{CE}}\left(A_{\mathrm{tm}}\right)}P_{\mathrm{CE}}r_{i}^{-\alpha }h_{i}G_{\mathrm{tm}}+\sum_{{\mathrm{CE}}_{i}\in \Theta_{\mathrm{CE}}\left(A_{\mathrm{ts}}\right)}P_{\mathrm{CE}}r_{i}^{-\alpha }h_{i}G_{\mathrm{ts}}\right)r_{j}^{-\alpha }h_{j}G_{\mathrm{rm}}G_{\mathrm{tm}}}\right]\\ \; & \overset{\left(k\right)}{=}\underset{\phi_{1}}{\underset{︸}{E\left[e^{-BP_{a}\eta \;\;\sum_{{\mathrm{CE}}_{i}\in \Theta_{\mathrm{CE}}\left(A_{\mathrm{tm}}\right)}\;\;\;\;P_{\mathrm{CE}}r_{i}^{-\alpha }h_{i}G_{\mathrm{tm}}G_{\mathrm{tm}}G_{\mathrm{rm}}r_{j}^{-\alpha }h_{j}}\right]}}\;\;\cdot \;\underset{\phi_{2}}{\underset{︸}{E\left[e^{-BP_{a}\eta \;\;\sum_{{\mathrm{CE}}_{i}\in \Theta_{\mathrm{CE}}\left(A_{\mathrm{ts}}\right)}\;\;\;\;P_{\mathrm{CE}}r_{i}^{-\alpha }h_{i}G_{\mathrm{ts}}G_{\mathrm{tm}}G_{\mathrm{rm}}r_{j}^{-\alpha }h_{j}}\right]}}, \end{array}$$

where (k) results from the fact that the two HPPPs of CEs distributed in regions $A_{\mathrm{tm}}$ and $A_{\mathrm{ts}}$ are independent with each other. Expression $\phi_{1}$ can be expressed as follows:

(A17) $$\begin{array}{cc} \phi_{1} & =E_{r_{i},h_{i},h_{j}}\left[\prod_{{\mathrm{CE}}_{i}\in \Theta_{\mathrm{CE}}\left(A_{\mathrm{tm}}\right)}e^{-BP_{a}\eta P_{\mathrm{CE}}r_{i}^{-\alpha }h_{i}G_{\mathrm{tm}}G_{\mathrm{tm}}G_{\mathrm{rm}}r_{j}^{-\alpha }h_{j}}\right]\\ \; & \overset{\left(l\right)}{=}E_{h_{j}}\left[E_{r_{i}}\left[\prod_{{\mathrm{CE}}_{i}\in \Theta_{\mathrm{CE}}\left(A_{\mathrm{tm}}\right)}E_{h_{i}}\left[e^{-BP_{a}\eta P_{\mathrm{CE}}r_{i}^{-\alpha }h_{i}G_{\mathrm{tm}}G_{\mathrm{tm}}G_{\mathrm{rm}}r_{j}^{-\alpha }h_{j}}\right]\right]\right]\\ \; & \overset{\left(m\right)}{=}E_{h_{j}}\left[\exp\left(-\frac{\lambda_{\mathrm{CE}}}{N}\int_{0}^{\theta_{\mathrm{tm}}}\int_{0}^{\infty }1-E_{h_{i}}\left[e^{-BP_{a}\eta P_{\mathrm{CE}}r_{i}^{-\alpha }h_{i}G_{\mathrm{tm}}G_{\mathrm{tm}}G_{\mathrm{rm}}r_{j}^{-\alpha }h_{j}}\right]r_{i}dr_{i}d\theta_{i}\right)\right]\\ \; & =E_{h_{j}}\left[\exp\left(-\frac{\lambda_{\mathrm{CE}}\theta_{\mathrm{tm}}}{N}\cdot \frac{\pi {\left(BP_{a}\eta P_{\mathrm{CE}}G_{\mathrm{tm}}G_{\mathrm{tm}}G_{\mathrm{rm}}h_{j}\right)}^{\frac{2}{\alpha }}r_{j}^{-2}}{\alpha \sin\frac{2\pi }{\alpha }}\right)\right]\\ \; & =\int_{0}^{\infty }\exp\left(-\xi \theta_{\mathrm{tm}}{\left(G_{\mathrm{tm}}G_{\mathrm{tm}}G_{\mathrm{rm}}\right)}^{\frac{2}{\alpha }}\right)e^{-h}dh, \end{array}$$

where $\xi =\frac{\lambda_{\mathrm{CE}}\pi {\left(BP_{a}\eta P_{\mathrm{CE}}h\right)}^{\frac{2}{\alpha }}r_{j}^{-2}}{N\alpha \sin(\frac{2\pi }{\alpha })}$, and (l) is obtained by the fact that $r_{i}$, $h_{i}$, and $h_{i}$ are mutually independent. Process (m) employs the PGFL property of a PPP: $E\left[\prod_{r\in \Theta }v\left(r\right)\right]=\exp\left(-\lambda {\;\int \int \;}_{S}\left(1-v\left(r\right)\right)rdrd\theta \right)$.

The expression $\phi_{2}$ can be derived by using a similar calculation process in Equation (A17) but by replacing $\Theta_{\mathrm{CE}}\left(A_{\mathrm{tm}}\right)$ with $\Theta_{\mathrm{CE}}\left(A_{\mathrm{ts}}\right)$, replacing $G_{\mathrm{tm}}G_{\mathrm{tm}}G_{\mathrm{rm}}$ with $G_{\mathrm{ts}}G_{\mathrm{tm}}G_{\mathrm{rm}}$, and replacing $\theta_{\mathrm{tm}}$ wtih $\theta_{\mathrm{ts}}$. Then, $\phi_{2}$ can be given by the following.

(A18) $$\begin{array}{c} \phi_{2}=\int_{0}^{\infty }\exp\left(-\xi \theta_{\mathrm{ts}}{\left(G_{\mathrm{ts}}G_{\mathrm{tm}}G_{\mathrm{rm}}\right)}^{\frac{2}{\alpha }}\right)e^{-h}dh. \end{array}$$

After inserting both Equations (A17) and (A18) into Equation (A16), we can have $\Phi_{1}$ as follows.

(A19) $$\begin{array}{c} \Phi_{1}=\int_{0}^{\infty }\exp\left(-\xi \theta_{\mathrm{tm}}{\left(G_{\mathrm{tm}}G_{\mathrm{tm}}G_{\mathrm{rm}}\right)}^{\frac{2}{\alpha }}\right)e^{-h}dh\cdot \int_{0}^{\infty }\exp\left(-\xi \theta_{\mathrm{ts}}{\left(G_{\mathrm{ts}}G_{\mathrm{tm}}G_{\mathrm{rm}}\right)}^{\frac{2}{\alpha }}\right)e^{-h}dh. \end{array}$$

After inserting $\Phi_{1}$ into Equation (A15), we can have $\Sigma_{1}$ given by the following.

(A20) $$\begin{array}{c} \Sigma_{1}=\exp\left(-\frac{\theta_{\mathrm{tm}}\theta_{\mathrm{rm}}\lambda_{\mathrm{BT}}}{2\pi N}\int_{0}^{\infty }\left(1-\Phi_{1}\right)r_{j}dr_{j}\right). \end{array}$$

Then, after using a similar calculation process of $\Sigma_{1}$, expressions $\Sigma_{2}$, $\Sigma_{3}$, and $\Sigma_{4}$ can be given by the following:

(A21) $$\begin{array}{c} \Sigma_{2}=\exp\left(-\frac{\theta_{\mathrm{ts}}\theta_{\mathrm{rm}}\lambda_{\mathrm{BT}}}{2\pi N}\int_{0}^{\infty }\left(1-\Phi_{2}\right)r_{j}dr_{j}\right). \end{array}$$

(A22) $$\begin{array}{c} \Sigma_{3}=\exp\left(-\frac{\theta_{\mathrm{tm}}\theta_{\mathrm{rs}}\lambda_{\mathrm{BT}}}{2\pi N}\int_{0}^{\infty }\left(1-\Phi_{3}\right)r_{j}dr_{j}\right). \end{array}$$

(A23) $$\begin{array}{c} \Sigma_{4}=\exp\left(-\frac{\theta_{\mathrm{ts}}\theta_{\mathrm{rs}}\lambda_{\mathrm{BT}}}{2\pi N}\int_{0}^{\infty }\left(1-\Phi_{4}\right)r_{j}dr_{j}\right). \end{array}$$

where $\Phi_{2}=\int_{0}^{\infty }\exp\left(-\xi \theta_{\mathrm{tm}}{\left(G_{\mathrm{tm}}G_{\mathrm{ts}}G_{\mathrm{rm}}\right)}^{\frac{2}{\alpha }}\right)e^{-h}dh\cdot \int_{0}^{\infty }\exp\left(-\xi \theta_{\mathrm{ts}}{\left(G_{\mathrm{ts}}G_{\mathrm{ts}}G_{\mathrm{rm}}\right)}^{\frac{2}{\alpha }}\right)e^{-h}dh$,

$\Phi_{3}=\int_{0}^{\infty }\exp\left(-\xi \theta_{\mathrm{tm}}{\left(G_{\mathrm{tm}}G_{\mathrm{tm}}G_{\mathrm{rs}}\right)}^{\frac{2}{\alpha }}\right)e^{-h}dh\cdot \int_{0}^{\infty }\exp\left(-\xi \theta_{\mathrm{ts}}{\left(G_{\mathrm{ts}}G_{\mathrm{tm}}G_{\mathrm{rs}}\right)}^{\frac{2}{\alpha }}\right)e^{-h}dh$, and

$\Phi_{4}=\int_{0}^{\infty }\exp\left(-\xi \theta_{\mathrm{tm}}{\left(G_{\mathrm{tm}}G_{\mathrm{ts}}G_{\mathrm{rs}}\right)}^{\frac{2}{\alpha }}\right)e^{-h}dh\cdot \int_{0}^{\infty }\exp\left(-\xi \theta_{\mathrm{ts}}{\left(G_{\mathrm{ts}}G_{\mathrm{ts}}G_{\mathrm{rs}}\right)}^{\frac{2}{\alpha }}\right)e^{-h}dh$.

Finally, after inserting both $\Sigma_{1}$ and $\Sigma_{2}$ into Equation (A13), inserting both $\Sigma_{3}$ and $\Sigma_{4}$ into Equation (A14), and combining with Equation (A12), we can obtain the Laplace transform $L_{I}\left(B\right)$ provided by Equation (14).

## Appendix E. Proof of Theorem 2

The spatial throughput can be expressed as follows:

(A24) $$\begin{array}{cc} T & =\lambda_{\mathrm{BT}}E\left[\log_{2}\left(1+\mathrm{SIN}R\right)\right]\\ \; & =\lambda_{\mathrm{BT}}\int_{0}^{\infty }P\left[\log_{2}\left(1+\mathrm{SIN}R\right)>t,\mathrm{SIN}R>\delta ,P_{\mathrm{BT}}^{r}\geq Q\right]dt\\ \; & =\lambda_{\mathrm{BT}}\int_{0}^{\infty }P\left[\mathrm{SIN}R>e^{t}-1,\mathrm{SIN}R>\delta ,P_{\mathrm{BT}}^{r}\geq Q\right]dt\\ \; & \overset{\left(n\right)}{=}\lambda_{\mathrm{BT}}\int_{\log_{2}\left(1+\delta \right)}^{\infty }P\left[\mathrm{SIN}R>e^{t}-1,P_{\mathrm{BT}}^{r}\geq Q\right]dt\\ \; & \overset{\left(o\right)}{=}\lambda_{\mathrm{BT}}\int_{\log_{2}\left(1+\delta \right)}^{\infty }P_{c}\left(e^{t}-1\right)dt, \end{array}$$

where (n) is obtained by letting $e^{t}-1>\delta$. Then, we can have $t>\log_{2}\left(1+\delta \right)$. Process (o) results from $P_{c}\left(e^{t}-1\right)=P\left(\mathrm{SIN}R\geq e^{t}-1,P_{\mathrm{BT}}^{r}\geq Q\right)$ according to Equation (11).

Then, after inserting Equation (15) into Equation (A24), the spatial throughput can be expressed by Equation (17).
